# Applications of machine learning in familial hypercholesterolemia

**DOI:** 10.3389/fcvm.2023.1237258

**Published:** 2023-09-26

**Authors:** Ren-Fei Luo, Jing-Hui Wang, Li-Juan Hu, Qing-An Fu, Si-Yi Zhang, Long Jiang

**Affiliations:** ^1^ Department of Cardiovascular Medicine, the Second Affiliated Hospital of Nanchang University, Nanchang, China; ^2^ Department of Clinical Medicine, Nanchang University Queen Mary School, Nanchang, China; ^3^ Department of Nursing, Nanchang Medical College, Nanchang, China

**Keywords:** familial hypercholesterolemia, machine learning, screening, diagnosis, risk assessment

## Abstract

Familial hypercholesterolemia (FH) is a common hereditary cholesterol metabolic disease that usually leads to an increase in the level of low-density lipoprotein cholesterol in plasma and an increase in the risk of cardiovascular disease. The lack of disease screening and diagnosis often results in FH patients being unable to receive early intervention and treatment, which may mean early occurrence of cardiovascular disease. Thus, more requirements for FH identification and management have been proposed. Recently, machine learning (ML) has made great progress in the field of medicine, including many innovative applications in cardiovascular medicine. In this review, we discussed how ML can be used for FH screening, diagnosis and risk assessment based on different data sources, such as electronic health records, plasma lipid profiles and corneal radian images. In the future, research aimed at developing ML models with better performance and accuracy will continue to overcome the limitations of ML, provide better prediction, diagnosis and management tools for FH, and ultimately achieve the goal of early diagnosis and treatment of FH.

## 1. Introduction

Familial hypercholesterolemia (FH) is a common autosomal dominant disease that is an inherited metabolic disorder (1). The main characteristic of FH is abnormally high levels of low-density lipoprotein cholesterol (LDL-C) in plasma, resulting in an increased risk of early-onset atherosclerosis and premature cardiovascular disease (1, 2). Heterozygous FH (HeFH) has a prevalence of 1 in 200–500 persons. Despite high incidence rate, the global diagnostic rate still remains low, and in most countries only 1% FH patients are diagnosed (3, 4). Homozygous FH (HoFH) is rarer but more severe, with an estimated prevalence of 1 in 300,000–360,000 persons, and it involves higher LDL-C levels and physical signs, such as the early presence of cholesterol deposits on the skin, eyes, and tendons (3, 5). Although there has been great progress in the study of FH, some challenges remain. For example, statins and other lipid-lowering therapies have been widely used, and the detection and treatment of FH is still unsatisfactory (1, 6). Missed diagnosis at an early age can lead to severe cardiovascular events (7, 8), but early lipid-lowering therapies can slow the onset of coronary heart disease in FH patients (4). The initiation of lipid-lowering therapy in FH patients in childhood slows the progression of atherosclerosis and reduces the incidence of cardiovascular events in adulthood (9). Therefore, it is important to diagnose and treat these patients early.

Artificial intelligence (AI) is a broad field that uses machines to imitate human behaviors, including their thought processes, learning abilities, and knowledge storage abilities (10, 11). One of the core parts of AI is machine learning (ML), which is a virtual branch of AI in medical applications (12). ML refers to the ability of computers to learn from experience, and ML models can use algorithms to detect patterns in a series of existing data they receive and train themselves to make predictions using new data (10, 13). In cardiovascular medicine, ML has emerged in many fields, such as disease prediction and diagnosis. For example, in a recent study, an ML-based race-specific model was developed to predict the risk of heart failure (14). Compared with performance of a model in which race is a covariate, new ML models have better performance (the C-index of the race-specific model for black adults is 0.88, and that of the nonrace-specific model is 0.81) (14). Another ML model (PRAISE score) was developed and proven to be feasible in predicting all-cause death, myocardial infarction, and major bleeding after acute coronary syndrome. In an external test, this model showed an area under the curve (AUC) of 0.92, 0.81 and 0.86 for these three events in one year, respectively (15). In addition, an algorithm with superior performance compared with that of a state-of-the-art algorithm was used to automatically classify heart disease by recognizing heartbeats with electrocardiogram signal features (16). In the future, AI will play a pivotal role in the field of cardiovascular diseases (11).

Recently, with the continuous research in ML in the cardiovascular field, various types of ML models have been progressively applied to FH. In this review, we provide an overview of the application of ML in FH screening, diagnosis, and risk assessment to help practicing clinicians and the general public understand the following core issues: (1) What progress has been made in the application of ML models in the field of FH diseases; (2) Advantages, challenges and prospects of ML model applications.

## 2. Methods

Relevant literature was searched through PubMed and Web of Science databases using the following search terms: “familial hypercholesterolemia,” “artificial intelligence,” and “machine learning.” We searched for articles prior to 2023.03 and selected only original research articles, excluding reviews, case reports, etc. References of relevant literature were also reviewed. Two independent evaluators reviewed the full text of the literature based on the following inclusion criteria: (1) The study population was patients with FH; (2) The results contain metrics for model performance, such as accuracy, sensitivity, specificity, and AUC etc.; (3) Comparisons of the performance of the ML models were made. Articles that did not address FH or use ML methods were excluded. The same two evaluators extracted study characteristics from the included articles, such as first author's name, age, study purpose, data source, sample size, algorithm type, and model performance metrics. Disagreements between evaluators were resolved through discussion. Selected literature was downloaded and merged into Endnote software (https://endnote.com/), removing duplicate papers.

## 3. Results

Thirty-one articles were identified based on our search strategy; article types and abstracts were screened for fit with key themes. Finally, a total of 18 papers were selected after confirmation of inclusion and exclusion criteria. All studies were categorized into three categories based on the type of application of ML to FH disease: screening, diagnosis, and risk assessment/other categories. Most of the studies (*n* = 13) focused on screening and diagnosis of FH. We will characterize the application of ML models according to this classification separately.

### 3.1. ML applied to the screening of FH

Traditional FH screening mainly relies on plasma lipid screening for specific high-risk populations, such as patients with early-onset arteriosclerotic cardiovascular disease (ASCVD) and those with a family history of FH or hyperlipidemia. Furthermore, cascade screening through genetic testing is commonly utilized. Nevertheless, the approach of selective screening has led to a significant rate of undetected diagnoses (17). In an attempt to screen for hyperlipidemia among children, a cholesterol assessment solely dependent on family history overlooked 9.5% of individuals with dyslipidemia (18). The presently recommended universal screening approach unavoidably introduces the challenge of being time-consuming. The application of machine learning in disease screening can automate the interpretation of results and may provide a more efficient and time-saving method for FH screening.

In FIND (Flag, Identify, Network, Deliver) FH project, an electronic health record (EHR) containing medication, diagnostic, procedure and laboratory examination data is used as the input to train the model (19, 20). Banda et al. (19) developed a classifier using EHRs from Stanford Health Care. A random forest (RF) classifier was trained with data from 197 confirmed FH patients and 6,590 matched non-case patients. The probability of FH for each patient output by the classifier was reviewed, and out of 56 predictions with a probability score of 0.90–0.99, 47 were identified as possible or clear FH after evaluation by Dutch Lipid Clinical Network (DLCN) and Make Early Diagnosis to Prevent Early Death (MEDPED) standards. This model showed good positive predictive value (PPV, 0.85) and sensitivity (0.68) in an external validation (466 cases, 5,000 non-case patients) from the Geisinger dataset, with an area under the receiver operating characteristic curve (AUROC) of 0.94, illustrating the excellent performance and practicability of the RF classifier. However, the different prevalence of FH in the data and the limited training dataset may have contributed to the differences in the classifier performance and certain limitations. In another FIND FH project, Myers et al. (20) used larger health care data to build a stochastic classifier model. In this work, the model was trained with data from 939 patients with confirmed FH and 83,136 individuals presumed to be free of FH. The results showed a PPV of 0.85, an area under the precision-recall curve of 0.55 and an AUROC of 0.89. In the external validation of the two cohorts, 1,331,759 individuals in a national dataset (*n* = 170,416,201) and 866 individuals in the Oregon Health & Science University dataset (*n* = 173,733) were flagged by the model as likely to have FH. Subsequently, FH experts reviewed 45 and 103 flagged individuals in two cohorts using four methods (the DLCN, MEDPED, Simon Broome, and clinical judgment by physicians), respectively. They found that 87% and 77% of the individuals were classified as probable or definite FH, respectively, demonstrating the accuracy and efficiency of the model. In an observational study, Sheth et al. prospectively implemented the FIND FH model to screen for FH (21). Based on EHRs from the University of Pennsylvania Healthcare System (*n* = 1,607,606), there were 8,614 patients with a FIND FH score >0.2, suggesting possible FH. Finally, 46 of 153 (30%) were diagnosed with FH, 31 of whom were newly diagnosed through either a physician clinical assessment, clinical diagnosis criteria or genetic testing. Although there is sometimes a significant gap between the low diagnostic rate in validation and the high predictability of FIND FH project, relying on this model to target screening for highly likely FH patients can significantly reduce misdiagnose rate of FH (Table 1).

**Table 1:** Studies of different algorithm models in FH.

Study	Objectives	Data sources	Age (Mean ± SD/Range)	Sample size	Algorithm/software	ML model performance
J. M. Banda et al. (19)	Detect FH using Stanford Health Care's EHR	Stanford Health Care	FH: Female: 47.8 ± 15.1, Male: 44.9 ± 13.9 Non-FH: Female: 50.4 ± 16.1, Male: 49.9 ± 14.2	12,253 patients	RF	Internal AUC 0.94, F1 Score 0.81, external AUC 0.94, F1 Score 0.75
K. D. Myers et al. (20)	Early diagnosis FH using EHR	Stanford University, University of Pennsylvania, Geisinger Medical Center, and Ohio State University	Training dataset: 35–79 external validation datasets: 30–76	170,674,009 patients	RF	AUC 0.89, PPV 0.85, sensitivity 0.45
S. Sheth et al. (21)	Detect FH using EHR	University of Pennsylvania Healthcare System	<75	8,614 patients	RF	46 (30%) were diagnosed with 31 were newly diagnosed
R. Hesse et al. (22)	Detect FH using basic lipid profile data	Laboratory information systems	Internal data set: Training data: FH+: 46 ± 17), FH−:50 ± 15 testing data: FH+: 47 ± 17), FH−:47 ± 15 external data set: High prevalence: FH+: 43 ± 13, FH−: 52 ± 12	6,851 patients	LR + DLM + RF	Internal AUC, external high prevalence AUC ML: AUC 0.754, AUC 0.711 LDL-C cut-off: AUC 0.682, AUC 0.642 DLCN: AUC 0.755, AUC 0.705
J. Gratton et al. (26)	Screening for FH variant carriers	The UK Biobank	51–63	139,779 participants	Regression	Internal AUC 0.78, external AUC 0.77,
T. Kocejko et al. (30)	Screening against potential FH by presence of CA	The National Centre of FH in Gdansk	27–58	3,900 images	CNN (VGG, ResNet and Inception)	Accuracy 0.88, F1 score 0.86
J. Albuquerque et al. (35)	Diagnosis FH	The Portuguese FH study	Medicated patients: FH: 47.3 ± 14.8, non-FH: 48.2 ± 13.0 non-medicated patients: FH: 33.7 ± 12.2, non-FH: 39.7 ± 10.8	451 individuals	LR, RF, XGB, NB	LR AUROC 0.84, RF AUROC 0.82, XGB AUC 0.82, NB AUC 0.81
J. Besseling et al. (36)	Predict FH causing mutation	The Dutch FH screening programme	HeFH patients: 38.1 ± 20.6, unaffected relatives: 43.1 ± 20	67,309 individuals	LR	Internal AUC 0.854, external AUC 0.954
S. F. Weng et al. (37)	Identifying patients with highest probability of FH	CPRD in the UK	Derivation cohort: men: 49 ± 15.9, women: 50 ± 17.4 Validation cohort: men: 49 ± 15.8, women: 50 ± 17.4	2,975,281 patients	FAMCAT, LR	AUC 0.860, sensitivity 0.70, specificity 0.88
R. K. Akyea et al. (38)	Assessed performance of machine-learning approaches for enhancing detection of FH	CPRD in the UK	>16	4,027,775 individuals	GBM, RF, ensemble learning, LR, deep learning	GBM AUC 0.892, RF AUC 0.891, ensemble AUC 0.890, LR AUC 0.812 deep learning 0.892
K. E. Niehaus et al. (39)	Identifying patients that may have FH	The Stanford Translation Research Integrated Database	/	1,013 patients	RF, LR	RF AUCROC 0.905, AUPRC 0.294 LR AUCROC 0.822, AUPRC 0.227
A. Pina et al. (41)	Diagnosis FH	Lipid clinics in Gothenburg (Sweden) and Milan (Italy)	Gothenburg: 38–60 Milan: 28–55	612 patients	GBM, NN, CT	Internal AUC: GBM 0.83, NN 0.83, CT 0.79 External AUC: GBM 0.78, NN 0.76, CT 0.70
A. Larrea-Sebal et al. (42)	Predicting the activity of missense LDLr mutations.	The ClinVar database	/	744 LDLr variants	MLb-LDLr	AUROC 0.932
L. Wang et al. (43)	FH risk assess	Peking Union Medical College Hospital	63.02 ± 11.44	5,597 patients	XGB + RF + SVM + BPANN	AUC _class [94.85 ± 0.47], AUC _prob [98.66 ± 0.27]
L. F. Reeskamp et al. (44)	Assess association between altered DNA Methylation and FH	Academic Medical Center in Amsterdam	FH mutation positive: 38.1 ± 12.0 FH mutation negative: 50.7 ± 12.3	136 patients	GBM	AUC 0.80 ± 0.17
A. Nemeth et al. (45)	Assess the associations of serum Lp(a) levels and ASCVD in FH	University of Debrecen Clinical Center's hospital information system	Non-FH: 22.2–59.0, FH: 39.3–59.7	590,500 patients	MLP + gradient boosting + SVM + binary linear regression	\

AUC, area under the curve; AUROC, area under the receiver operating characteristic curve; AUPRC, area under the precision-recall curve; ASCVD, arteriosclerotic cardiovascular disease; BPANN, back-propagation artificial neural network; CA, corneal arcus; CNN, convolutional neural networks; CPRD, clinical practice research data link; CT, classification tree; DLCN, Dutch lipid clinic network; DLM, deep learning model; EHR, electronic health record; FAMCAT, familial hypercholesterolaemia case ascertainment tool; GBM, gradient boosting machine; LR, logistic regression; LDLr, low-density lipoprotein receptor; MLb-LDLr, machine learning–based LDLr predictive software; Lp(a), lipoprotein (a); MLP, multilayer perceptron; NB, naive bayes; NN, neural network; PPV, positive predictive value; RF, random forest; SVM, support vector machine; XGB, extreme gradient boosting.

In line with EHR, machine learning models built on basic lipid data training can also be applied to disease screening. Hesse et al. (22) used the primary lipid profile data [Total Cholesterol (TC), High-density lipoprotein cholesterol, LDL-C and Triglycerides] from the laboratory information systems (*n* = 555, 68% White individuals, 26% Indian individuals, and 3.2% Black African individuals) to create an ML model that combined logistic regression (LR), deep learning, and RF classification algorithms. In this study, patients with blood lipid levels exceeding LDL-C cutoff (4.5 mmol/L) and a model labeled probability of disease greater than 60% were identified as likely or clear FH. This model was trained on 70% of the internal data sets, and outperformed the LDL-C threshold in both the 30% internal validation set test (AUROC 0.754 vs. 0.682) and the external validation (AUROC 0.711 vs. 0.642) of the Groote Schuur Hospital database (*n* = 1,376; FH prevalence = 64%), showing better performance and accuracy. In addition, the accuracy and F score of the model were higher in the medium and low prevalence cases with AUROC curve values of 0.801 and 0.856, respectively (22). Therefore, based on simple lipid spectrometry data, the ML model still accurately identifies FH patients and has better screening performance than the LDL-C cutoff value.

Regardless of the variables considered, the combinatorial nature of variable selection in ML model construction brings about model diversity. Changes in LDL-C levels in patients with FH can be attributed to either a single pathogenic genetic variant or a combination of multiple variants. The polygenic score (PGS) encompasses genetic variation information and has the capability to predict LDL-C levels. Severe polygenic hypercholesterolemia patients exhibit elevated PGS values (23). Serving as a valuable adjunct to FH sequencing techniques, PGS enables differentiation between monogenic FH and non-monogenic hyperlipidemia (24). Specifically, the frequency of polygenic anomalies is notably greater among adult FH patients when contrasted with pediatric patients. Consequently, leveraging PGS data could enhance the accuracy of identifying these individuals (25). Nevertheless, the integration of PGS data into clinical practice is not yet a commonplace occurrence. In Gratton et al.'s study, PGS of LDL-C was included as a predictor variable for the first time (26). Cohort data were obtained from the UK Biobank and included 139,779 (488 FH variant carriers and 139,291 non-carriers) participants of white ancestry who had undergone whole-genome sequencing. Two (14 and 9 variables, respectively) multivariate ML models were constructed based on a least absolute shrinkage and selection operator (LASSO) regression algorithm. The former, which retained LDL-C PGS and other variables such as statins, lipid data and clinical information, obtained AUCs of 0.78 on the training set (80% of the data set) and 0.77 on the test set (20% of the data set), respectively. The latter obtained an AUC of 0.76 on the test set. In tests to predict pathogenic variants of FH, the LASSO with PGS model still performed well in predicting pathogenic variants of APOB (AUC = 0.81) and LDLR (AUC = 0.76). In a screening assessment of 100,000 individuals, this model (threshold = 0.6%) recommended 18% fewer genetic tests compared to the LDL-C and statin use model (12,033 vs. 14,700). Overall, this multivariate ML model for detecting FH variant carriers outperforms the common LDL-C-based model and may reduce the burden of gene sequencing in future FH screening efforts.

Finally, an ML model can also rely on data obtained from corneal arcus detection to achieve FH screening. The presence of corneal arcus (CA) often indicates lipid abnormalities and provides strong physical evidence for screening patients at high risk for FH (27). In a study of early-onset CAD with CAs, potential FH patients had a CA incidence of 55.31%, which was as high as 90% in confirmed patients, reflecting the close relationship between the occurrence of CA and FH (28). Traditional methods of identifying CA rely on the interpretation of iris images by a medical professional, which is time-consuming and subject to interpretation discrepancies (29). To this end, Kocejko et al. (30) designed a mobile application based on a convolutional neural network (CNN) model to identify CA. The training data consisted of 3,900 iris images of various stages of CAs and iris images without CAs, mainly from the University Clinical Centre Gdansk. The authors trained and tested three different CNN models and further tested them separately with black and white masked datasets. When using a dataset simulating a “real life” scenario, an accuracy of 0.88 and an F1-score of 0.86 were obtained with a model assessed with white masked images, with better results than those with black masks. This application provides a new, faster and more accurate way to identify CAs. In this way, the screening of FH could benefit from the screening of clinical features.

### 3.2. ML applied to the diagnosis of FH

There are approximately 20 million patients with FH worldwide, 90% of whom are underdiagnosed, and untimely and inadequate diagnosis seriously affects the prognosis of the disease (31). There are no uniform criteria for the diagnosis of FH, and the most commonly used criteria in clinical practice are the DLCN criteria (9, 32) and the Simon Broome criteria (33). However, these criteria have several limitations, such as an imbalance of high sensitivity and low PPV, and the absence of information such as clinical history and family history makes diagnosis difficult. The gold standard for the diagnosis of FH is genetic testing (34). However, due to the high cost and lack of reliable evaluation of whether a new mutation is pathogenic, it cannot be widely promoted, especially in low-income countries. Therefore, it is necessary to improve existing diagnostic methods or use powerful auxiliary diagnostic tools to achieve more reliable diagnosis for FH.

As demonstrated in the previous FINDFH study (22), the ML models have recognition performance comparable to DLCN criteria in internal validation (*n* = 166, AUROC 0.754 vs. 0.755) and external validation of high prevalence (64%) (*n* = 1,376, AUROC 0.711 vs. 0.705), suggesting that ML models can replace clinical criteria and provide new insights for future FH diagnostics. However, different algorithmic models exhibit performance differences in identifying FH, which reflects the importance of algorithmic model selection when developing alternative diagnostic procedures (35). In previous studies, the LR model performed well in identifying FH cases (AUROC >0.8) (36, 37), but the RF model performed better in the studies of Akyea et al. (38) (0.89 vs. 0.81) and Niehaus et al. (39) (0.905 vs. 0.822). Recently, Albuquerque et al. (35) combined different algorithms (naive Bayes classifier, LR, RF and extreme gradient boosting) with the synthetic minority oversampling technique (SMOTE) or maximizing Youden index (YI) and performed comparative analysis. The sample for this study was derived from the Portuguese FH study. Serum TC and LDL-c values were used as the primary included variables, with other laboratory tests, biological and clinical information as candidate predictor variables. Data from the 451 individuals in the model dataset included 334 medicated patients (*n* = 111, molecular diagnosis positive) and 117 nonmedicated patients (*n* = 35, molecular diagnosis positive). The results showed that the LR model performed best (0.84 AUROC and 0.71 Area Under the Precision-Recall Curve) regardless of the data processing technique used to address the classification imbalance problem, and the performance was maintained. The accuracy, G-mean, and F1 scores of all classification methods were higher than those of the Simon Broome criterion, representing higher classification efficiency and more balanced recognition capabilities (35). The superiority of SMOTE for model interpretation makes its combination with the LR model more concise, and it is recommended for FH identification. However, more studies that compare and improve ML-based automatic diagnosis methods and apply reasonable data processing techniques to improve the recognition level of FH are needed (40).

ML models can also be applied to predict pathogenic mutations in FH to provide a “virtual” genetic diagnosis. Pina et al. (41) used three machine learning algorithms (classification tree (CT), gradient boosting machine (GBM), and neural network (NN)) to predict the presence of FH-causing mutations in the Gothenburg cohort (*n* = 248,111 mutation-positive) and the Milan cohort (*n* = 364 with 307 mutation-positive). With an internal test of the Gothenburg cohort (*N* = 74), NN achieved the best performance, with a mean AUROC of 0.83. With an external test of the Milan cohort (*N* = 364), GBM performed best, with a mean AUROC of 0.779. In addition, in the internal cohort, NN and GBM have PPV and NPV greater than 0.75. In the external cohort, NPV is lower for all algorithms (cut-off 0.5) and Dutch Lipid Score (>6 points).In both tests, NN and GBM performed comparably and better than CT overall, and different algorithms performed better than DLCN standard scores (average AUROCs of 0.683 and 0.64 for the external and internal tests). Collectively, the algorithmic model showed better expressiveness than Dutch Lipid Score in detecting gene mutations. In another study, Larrea-Sebal et al. (42) developed an ML model-based software called ML-based LDL receptor software (MLb-LDLr) for predicting missense LDLr mutations of pathogenicity. In this study, data for training (499 pathogenic and 54 benign variants) and validation (166 pathogenic and 26 benign variants) were obtained from the ClinVar database, and the model prediction accuracy exceeded 90% for both pathogenic and benign variants during training and validation. When validated using all missense variants from the ClinVar database (*n* = 744), 60% of the variants were able to be identified by the Mlb-LDLr optimized through Excel Solver Evolutionary algorithm strategy, and an AUROC of 0.932 was obtained. The accuracy of Mlb-LDLr was ultimately validated by functional prediction of 13 undetermined LDLR variants in ClinVar. The software can achieve good accuracy and excellent balance in detecting pathogenic (72%, *n* = 11) and benign variants (50%, *n* = 2), illustrating that it can effectively help predict known pathogenicity in FH mutation. With the rapid development of gene detection technology, a large number of unknown LDLr variants have been detected and discovered, and a novel ML based predictive model software for predicting the pathogenicity of LDLr variants can be used as a practical auxiliary tool to effectively help clinicians in the diagnosis of FH.

### 3.3. ML applied to risk assessment of FH and beyond

Apart from applications in FH screening and diagnosis, ML has also been used in risk assessment.

Aiming to develop a risk assessment method based on Chinese patients with ASCVD, Wang L et al. developed and evaluated a hybrid FH risk assessment tool (HFHRAT), a combination of three FH risk assessment tools and stacking models (43). To develop this tool, two risk assessment tools, modified DLCN for China (mDLCN) criteria and the Taiwan (TW) criteria (Supplementary Material), had the best performance among the 10 tools (the mDLCN criteria had a higher sensitivity and specificity of 97.22% and 92.90%, respectively, and the Taiwan criteria had the highest specificity of 100%) using the DLCN criteria as the reference (44, 45). The selected criteria as well as the DLCN criteria were combined with a voting strategy to generate a novel tool, and the predictor setting dataset was divided by the hybrid result (HYR) into 1,112 high-risk and 4,485 low-risk participants. In a further development of this tool, nine variables and HYR were used, and the stacking models had the best performance with AUC_class [94.85 ± 0.47] and AUC_prob [98.66 ± 0.27] (40). In the interpretation of HFHRAT, it was suggested from the individual conditional expectation (ICE) and partial dependence plot (PDP) that individuals aged <75 years with LDL-c >4 mmol/L were more likely to exhibit FH. In addition, comparing the predictive characteristics of the five tools, HFHRTA could adjust the position of the median of the data, resulting in a lower false negative rate than existing tools, indicating that this hybrid tool has a higher ability to predict high-risk FH patients (43). The research and improvement of such risk assessment tools will also be able to play a role in the screening and diagnosis of FH, effectively improving the prognosis of the disease.

In addition, ML was used to assess the relationship between FH and other risk factors, such as altered DNA methylation and serum Lp(a) levels (46, 47). In this study, data from DNA methylation measurements were analyzed with linear regression models and gradient boosting machine learning in two steps. The gradient boosting model had an average AUC of 0.80 ± 0.17 in 50 repeat tests of distinguishing methylation differences in FH mutation-negative and FH mutation-positive patients (46). In another study, ML models were trained to identify FH patients in the Hungarian population, and it was found that serum Lp(a) levels and the frequency of atherosclerotic complications were much higher in FH patients, but there were no significant associations between serum Lp(a) levels and atherosclerotic vascular diseases in the Hungarian FH patient group (47).

## 4. Challenges

ML applications in FH still face many challenges. The most pressing issue is the performance stability of the algorithm model. The algorithm models used have achieved excellent results in various application scenarios, but the adaptability of the algorithm models based on different datasets to different populations and different disease stages may vary. Therefore, future research should not only improve the model performance but also consider the generalizability. Second, most of the training and validation of ML models mainly rely on clinical data in EHRs and lack relevant research on image data, biological data and so on. Therefore, it is necessary to integrate multisource data, increase data volume, and enhance the credibility of ML models. Finally, there is still a lack of real external validation and application of algorithm models, and their popularization in real-world examples is difficult. On the one hand, this is due to the complexity of ML internal mechanisms that make it difficult to explain, reducing its credibility. On the other hand, regulatory and quality control issues encountered in the real application process also hinder its popularization. Despite these problems, we believe that with the deepening of follow-up research, suitable solutions will be found to successfully apply high-performance algorithm models to real clinical scenarios.

This review has some limitations. First, we conducted a literature search in only two databases (PubMed and Web of Science) which may cause bias and omissions in the selection of literature. Future reviews will expand searches to include larger databases and multiple language options for more comprehensive and diverse information. Second, our analysis did not go into the detailed analysis and comparison of different algorithms and parameters, and future research may key in finding the optimal algorithm among different algorithms for FH scenarios. Finally, the process of data extraction and analysis in our data may be limited by a number of factors such as the completeness, clarity, and availability of data from different original studies. In addition, there are differences in methodological choices for different data extraction and analysis, which may lead to biased interpretation of the results. Please suggest strategies for future improvement.

## 5. Conclusion

**Figure 1:**
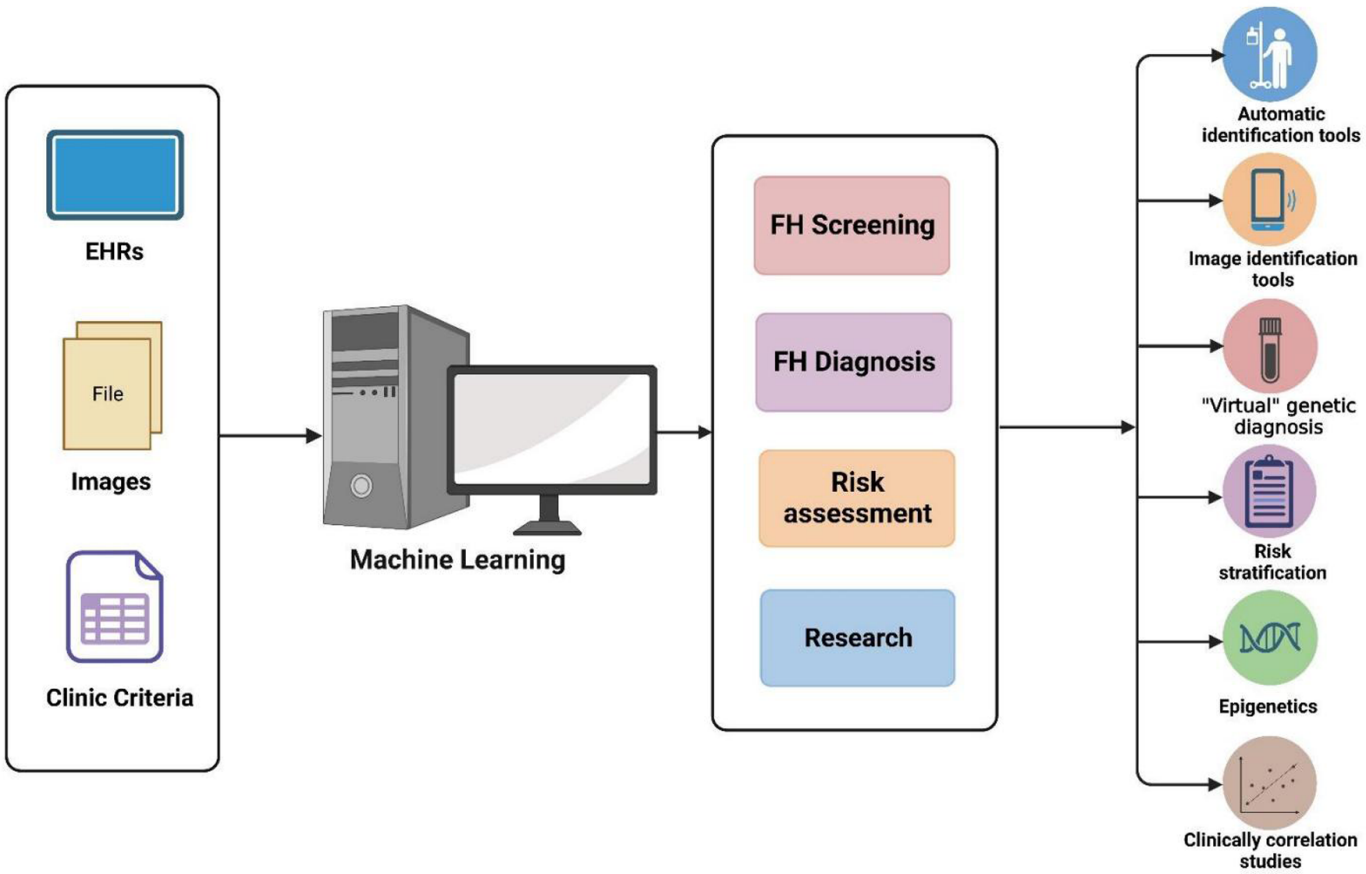
Applications of machine learning in FH.

To facilitate enhanced comprehension and practical adoption of the ML approach in managing FH disease, we present a comprehensive overview of studies that have employed ML for FH disease applications (Figure 1) and summarize the characteristics of each studied model in the form of a table (Table 1). This review allows us to recognize that (1) **Data Integration for Enhanced Outcomes:** ML models exhibit the capacity to effectively amalgamate diverse datasets including EHR, lipid profiles, PGS, CA results, and genetic test reports. This integration augments the accuracy of FH screening, diagnosis, and risk assessment processes; (2) **Comparative Superiority of ML Models**: Emerging research consistently showcases the potential of ML models to either match or surpass conventional clinical approaches founded on traditional criteria and LDL-C thresholds; (3) **Influential Performance Factors**: The performance of these models hinges upon factors such as the consideration of variables, sample size, algorithm selection, and utilization of distinct data-processing methodologies.; (4) **Balancing Advantages and Challenges**: While ML-driven disease management holds promise and substantial applicability, its effective execution presently grapples with noteworthy challenges..

Overall, the strategy of constructing ML models provides new ideas for solving important disease problems (48). In addition, with the continuous development of ML, better algorithmic models combined with mature data processing techniques will gradually eliminate the defects of the models themselves and further improve the model performance. At the same time, the “black box” problem caused by the internal complexity of ML algorithms can be addressed, and the trustworthiness of these applications to solve medical problems can be improved so that ML models can be more widely used. ML models will not be limited to integrating large volumes and multidimensional data such as clinical, genetic, and laboratory data to aid in disease screening and diagnosis, but will also penetrate the disease practice areas in a variety of forms. AI and ML will be able to help improve the quality of experiments and speed up the process of clinical trials, as well as simulate the outcome of treatments by intelligently generating “patients” in order to improve drug development and treatment of FH diseases (49). Recently, a chat tool named chat generative pretrained transformer (Chat-GPT), which is based on natural language models, has been developed to integrate rich medical data to provide “complete and accurate” medical information in medical queries (50). In the future, there may be more powerful medical assistant robots, which can not only play the role of teachers in medical education, but also create realistic simulation for different patient encounters to provide professional disease guidance (49). In conclusion, the advancing capabilities of AI and ML are poised to empower healthcare professionals through collaborative engagement. This synergy not only enhances the proficiency of healthcare practitioners but also facilitates more accessible human-to-human interactions. By fostering an interactive collaboration between computer scientists, clinicians, and patients, this model holds the potential to effectively tackle the health challenges encountered by FH patients.
