# Supplementary material for: Applications of machine learning in familial hypercholesterolemia
Source: Front Cardiovasc Med. 2023 Sep 26;10:1237258. doi: 10.3389/fcvm.2023.1237258 (PMC10562581; doi:10.3389/fcvm.2023.1237258)
Supplement: Supplementary file 1 [file Table1.docx]

**Applications of machine learning in familial hypercholesterolemia**

Ren-Fei Luo^#1^, Jing-Hui Wang^#1,2^, Li-Juan Hu^3^, Qing-An Fu^4^, Si-Yi Zhang^5^，Long Jiang^1^*

**Supplementary Data**

**Table S1. Modified DLCN definition for FH**

**Table S2. The FH diagnostic criteria of Taiwan**

**Table S1. Modified DLCN definition for FH**

| **Modified DLCN for China**   **points** | |
| --- | --- |
| **Family history** | |
| First-degree relative with known premature (men: <55 years; women:  <60 years) coronary artery disease or vascular disease. | 1 |
| **Clinical history** | |
| Patient with premature (men: <55 years; women: <60 years) coronary  artery disease | 2 |
| Patient with premature (men: <55 years; women: <60 years)  cerebrovascular or peripheral vascular disease | 1 |
| **Physical examination** | |
| Tendinous xanthomata | 6 |
| Arcus cornealis prior to age 45 years | 4 |
| **LDL-C levels** | |
| LDL-C ≥ 6 mmol/l (∼230 mg/dl) | 8 |
| LDL-C 5.0–5.9 mmol/l (∼190–29 mg/dl) | 5 |
| LDL-C 3.5–4.9 mmol/l (∼135–189 mg/dl) | 3 |
| LDL-C 2.5–3.4 mmol/l (∼97–134 mg/dl) | 1 |
| **DNA analysis** | |
| Causative mutation in the LDLR, ApoB or PCSK9 gene | 8 |
| >8 points Definite FH | |
| 6–8 points Probable FH | |
| 3–5 points Possible FH | |
| 0–2 points Unlikely FH | |

ApoB apolipoprotein B, DLCN Dutch Lipid Clinic Network, FH familial hypercholesterolemia, LDL-C Low-Density Lipoprotein Cholesterol, LDLR low-density lipoprotein receptor, PCSK9 Proprotein Convertase Subtilisin/Kexin Type 9

**Table S2. The FH diagnostic criteria of Taiwan**

| **Taiwan FH diagnostic criteria** | **points** |
| --- | --- |
| **Family history** | |
| First-degree relative with early vascular/coronary disease (male <45 y, female <55 y) OR Adult first-degree relative with LDL-C > 160 mg/dL | 1 |
| First-degree relative with xanthoma and/or corneal arcus OR First-degree relative <18 y with LDL-C > 130 mg/dL | 2 |
| **Clinical history** | |
| Patient with early coronary artery disease (male <45 y, female <55 y) | 2 |
| Patient with early cerebral or peripheral arterial disease (male <45 y, female <55 y) | 1 |
| **Physical examination** | |
| Xanthoma | 6 |
| Corneal arcus (<45 y) | 4 |
| **Level of LDL-C (mg/dL)** | |
| ≥330 | 8 |
| 250–329 | 5 |
| 190–249 | 3 |
| 155–189 | 1 |
| **DNA analysis** | |
| Presence of functional mutation of LDL-R, ApoB-100, or PCSK9 gene | 8 |
| **Diagnostic of FH** | |
| Definite FH | >8 |
| Probable FH | 6–8 |
| Possible FH | 3–5 |

ApoB-100 Apolipoprotein B-100, FH familial hypercholesterolemia, LDL-C Low-Density Lipoprotein Cholesterol, LDLR low-density lipoprotein receptor, PCSK9 Proprotein Convertase Subtilisin/Kexin Type 9
